# The Role of the Six-Minute Walk Test in the Functional Evaluation of the Efficacy of Rehabilitation Programs After COVID-19

**DOI:** 10.3390/life14111514

**Published:** 2024-11-20

**Authors:** Diana-Lidia Tache-Codreanu, Lucian Bobocea, Iuliana David, Claudia-Camelia Burcea, Corina Sporea

**Affiliations:** 1 Medical Rehabilitation Department, Colentina Clinical Hospital, Stefan cel Mare Street No. 19–21, 020125 Bucharest, Romania; bobocea.lucian95@gmail.com (L.B.); julexim@gmail.com (I.D.); 2 Faculty of Midwifery and Nursing, University of Medicine and Pharmacy “Carol Davila”, 37 Dionisie Lupu Street, 020021 Bucharest, Romania; corina.sporea@gmail.com; 3 National University Center for Children Neurorehabilitation “Dr. Nicolae Robanescu”, 44 Dumitru Minca Street, 041408 Bucharest, Romania

**Keywords:** post-COVID rehabilitation, respiratory recovery, Six-Minute Walk Test, physical therapy, kinesiotherapy, high-intensity laser, HILT

## Abstract

Patients with COVID-19 suffering in the acute phase from both the sequelae of the disease and prolonged immobilization require a rehabilitation phase for functional recovery and a comprehensive functional evaluation. This study proposes using the 6-Minute Walk Test (6MWT) as a global functional assessment tool to quantify rehabilitation outcomes in post-COVID patients. Additionally, investigating the effect of High-Intensity Laser Therapy (HILT) on patients with musculoskeletal comorbidities was another key research question. Two post-COVID rehabilitation programs were retrospectively analyzed as follows: one consisting of kinesiotherapy combined with High-Intensity Laser Therapy for patients with musculoskeletal comorbidities and the other consisting of kinesiotherapy alone. Functional evaluation using 6MWT was conducted before and after 10 daily therapeutic sessions with 33 ambulatory patients divided into 2 groups (18 patients treated with HILT and kinesiotherapy vs. 15 patients treated with kinesiotherapy only). The 6MWT was successfully completed by 32 out of 33 patients (96.96%), with performance improvements ranging from 3% to 60% among patients. Statistical differences were also observed between the groups, suggesting that the 6MWT is a sensitive, objective, and valuable tool for functional assessment of post-COVID rehabilitation, supporting the potential benefits of HILT in enhancing functional recovery.

## 1. Introduction

The COVID-19 pandemic, caused by the SARS-CoV-2 virus, has impacted millions of people globally, with significant consequences for healthcare systems and populations alike. As of 2019, over 80% of the world’s population has been affected by the virus through direct infection or by the overwhelming pressures on the healthcare infrastructure [1,2]. COVID-19 has demonstrated a wide range of effects on the human body, from mild respiratory symptoms to severe complications such as pneumonia [3], acute respiratory distress syndrome (ARDS) [4], and multi-organ failure [5]. Moreover, its long-term effects, now commonly referred to as post-COVID or “long COVID”, have brought to light the necessity of comprehensive rehabilitation programs [6,7].

For patients with pre-existing chronic conditions, such as diabetes [6,8], polyneuropathies [9]; stroke [5,10,11,12,13]; and rheumatic [14], degenerative [15], or oncological diseases [16,17], the pandemic posed an additional challenge. Limited access to healthcare services during the lockdowns and restrictions significantly affected the continuity of care for these individuals [18]. Regular medical interventions, such as kinesiotherapy and rehabilitation services, were often delayed or interrupted, exacerbating their underlying conditions. As a result, many patients faced worsened mobility, increased pain, or decreased overall functionality, which not only affected their physical health but also diminished their quality of life. Quality of life represents an individual’s perception of their existence, encompassing both the physical integrity and functionality of the body and their ability to perform tasks and engage in life situations [19,20,21,22,23,24,25].

Given the multifaceted impact of COVID-19 on both previously healthy and chronically ill patients, the rehabilitation phase has become a critical component in restoring functional capacity and overall quality of life. Assessing the efficacy of post-COVID rehabilitation programs is essential, especially for patients with pre-existing conditions, as they often require tailored interventions.

The Six-Minute Walk Test (6MWT), a well-established tool for measuring functional capacity and endurance [26,27], is a valuable method for evaluating the outcomes of these rehabilitation programs. The 6MWT is widely utilized across a range of pathological conditions. It has been successfully applied in studies involving arthritis and joint conditions [28,29,30], multiple sclerosis [31], pain management [32], Alzheimer’s disease [33,34], Parkinson’s disease and movement disorders [35,36], spinal cord injury [37], stroke recovery [38,39], spinal muscular atrophy [40,41], rehabilitation following lung transplant [42] and cardiac disorders [43], and in older adults and geriatric care [44,45,46].

This study hypothesized that post-COVID rehabilitation, particularly with the addition of High-Intensity Laser Therapy (HILT), would improve patient health and quality of life by enhancing effort tolerance, thereby supporting reintegration into society. To objectively assess these outcomes, we used the 6-Minute Walk Test (6MWT) to measure effort tolerance and walking capacity. The study examined the effectiveness of this test as a functional assessment tool in post-COVID rehabilitation by analyzing the impact of rehabilitation programs, including HILT, on 6MWT results and correlating these with clinical and biological indicators to highlight the potential benefits of HILT. Additionally, this study focused on patients with pre-existing chronic conditions to better understand the effectiveness of rehabilitation interventions in enhancing their physical capabilities and overall quality of life.

## 2. Materials and Methods

This retrospective study included 33 patients infected with the coronavirus who were presented to the Rehabilitation Department of Colentina Medical Hospital in Bucharest, Romania, for post-COVID evaluation and day hospitalization for specialized rehabilitation treatment between 2021 and 2023. The group consisted of men and women, aged between 30 and 80, with compensated mild to moderate multi-organ damage, respiratory conditions, and musculoskeletal disorders, namely dorsolumbar discopathy, knee osteoarthritis, and plantar fasciitis.

Inclusion Criteria:

- Age between 18 and 80 years;
- History of COVID-19, presented to the department 3 to 6 months after confirmed infection (via PCR or rapid antigen test), exhibiting post-viral symptoms, such as fatigue, decreased exercise tolerance, reduced global muscle strength, and exertional dyspnea, impacting functional walking tests;
- Stable patients with the ability to move (with or without a cane);
- Subjects evaluated at admission for biological laboratory analyses including the following: 25-hydroxy Vitamin D (25 OH Vit D), total calcium (total Ca), creatine kinase (CK), creatinine, D-dimers, fibrinogen, glucose, glycated hemoglobin (glycosylated HGB), leukocytes, hematocrit, erythrocytes, platelets, hemoglobin, C-reactive protein (PCR), total proteins, alanine aminotransferase (TGP), aspartate aminotransferase (TGO), urea, erythrocyte sedimentation rate (ESR), imaging (chest radiography), and functional perspectives (6MWT with dyspnea evaluation using the Borg scale, blood pressure, pulse, and oxygen saturation);
- Subjects who followed a complex rehabilitation treatment program and were re-evaluated functionally using the same tests/scales at admission for comparison.
- Exclusion Criteria:
- Age below 18 years or above 80 years;
- Incomplete data for evaluations specified within the inclusion criteria (biological, imaging, and functional assessments);
- Abnormal clinical evaluation values at admission, including blood pressure, pulse, and oxygen saturation at rest;
- Coexisting diseases affecting various systems that prevent the full implementation of the prescribed rehabilitation program or result in non-compliance with the treatment.

Demographic data and information on comorbidities—pulmonary damage (post-COVID pneumonia with mild or moderate lung involvement, secondary pulmonary fibrosis), cardiac involvement (therapeutically controlled hypertension, NYHA class 2 heart failure with preserved ejection fraction, therapeutically controlled sinus tachycardia), neurological involvement (such as discogenic radiculopathies and polyneuropathies), and musculoskeletal involvement (discopathies, functionally decompensated knee osteoarthritis, and plantar fasciitis)—were also collected.

### 2.1. The 6-Minute Walk Test (6MWT)

The 6MWT is a sub-maximal exercise test commonly used to assess walking endurance and aerobic capacity. It measures the distance a patient can walk on a flat, hard surface in six minutes. The test provides valuable information about functional status, especially in patients recovering from illness or with chronic conditions. As a simple and non-invasive tool, the 6MWT is widely used in clinical settings to monitor progress, especially in rehabilitation programs, where it helps quantify improvements in functional capacity over time [47]. Additionally, any symptoms that lead to the stopping of the test are assessed, offering further insight into a patient’s limitations [48].

This test calculates the ideal walking distance based on age, sex, weight, and height [47,49,50,51]. The formulas used are as follows:

- For men: 6 MWT (meters) = 7.57 × Height (cm) − 5.03 × Age (years) − 1.76 × Weight (kg) − 309
- For women: 6 MWT (meters) = 2.11 × Height (cm) − 5.78 × Age (years) − 2.29 × Weight (kg) − 667

The yield of the 6MWT was calculated with the following percentage ratio: (walking distance at discharge − walking distance at admission)/(ideal distance − walking distance at admission).

Before and after the test, blood pressure and pulse were measured using a sphygmomanometer, and oxygen saturation was assessed with a pulse oximeter. Additionally, the Borg scale for dyspnea and perceived exertion was used as part of the assessment.

### 2.2. The Borg Scale

The Borg scale is a widely used tool to measure perceived exertion during physical activity. It allows individuals to rate how hard they feel they are working, taking into account their overall effort, physical fatigue, and breathlessness. There are two common versions of the scale: the original 6–20 scale, which correlates with heart rate (e.g., a rating of 15 approximates a heart rate of 150 bpm), and the 0–10 scale, which is often used to assess symptoms like breathlessness. The Borg scale is particularly useful in clinical settings where objective measures of exertion may be less reliable, such as in patients on beta blockers [52,53,54,55].

After applying the inclusion and exclusion criteria, 33 post-COVID patients were selected. The patients were divided into two subgroups (18 patients versus 15 patients) based on the presence or absence of musculoskeletal disorders who followed different rehabilitation programs according to the following pathology:

- Subgroup 1 consisted of 18 patients with musculoskeletal disorders who underwent a rehabilitation program that included High-Intensity Laser Therapy (HILT) and physiotherapy;
- Subgroup 2 consisted of 15 patients without musculoskeletal disorders who received only physiotherapy as part of their rehabilitation program.

The rehabilitation programs included kinesiotherapy +/− HILT.

High-Intensity Laser Therapy (HILT) was applied using various protocols to different regions (paravertebral dorsolumbar, knee, or plantar), with specific parameters: total energy between 1300 J and 2175 J, with a total dose between 52 J/cm^2^ and 87 J/cm^2^, surface area of 25 cm^2^, time between 04:33 and 07:32 min, maximum power of 30.0 W, and average power between 3.2 W and 7.2 W, in 4 therapy sections (first two sections were for analgesic effect and the other sections were for biostimulation).

HILT was selected in our rehabilitation department for treating patients with musculoskeletal disorders post-COVID due to its three key benefits: analgesia; biostimulation leading to the regeneration of musculoskeletal tissues, including peripheral nerve regeneration; and its documented effect on the resorption of residual pulmonary lesions in post-COVID pneumonia when applied to the dorsal region, particularly in cases associated with dorsalgia [56,57]. The action mechanism of High-Intensity Laser (HIL) therapy includes physiological, anti-inflammatory, thermal, and clinical effects. Physiologically, HIL therapy enhances oxygen delivery, stimulates intracellular enzyme activity and DNA synthesis, activates the Na/K membrane pump to support metabolic functions, and modulates levels of local histamines, prostaglandins, and endorphins. Its anti-inflammatory effects occur through immune cell stimulation, reducing prostaglandin (PGE2) levels, and increasing prostacyclin (PGI2) synthesis. HIL therapy also promotes nerve repair by stimulating Schwann cells. The thermal effects of HIL induce muscle relaxation and provide analgesia to trigger points. Clinically, it has biostimulatory, anti-inflammatory, anti-edematous, vasodilatory, and pain-relieving effects. HILT is free of adverse effects and has proven beneficial across a range of conditions. In plantar fasciitis, it effectively reduces pain and enhances quality of life by improving functionality. It is also effective in managing knee osteoarthritis and back pain, and in reducing disability associated with lumbar sciatica accompanied by motor deficits, particularly in patients with musculoskeletal and neurological comorbidities, thereby improving gait. Furthermore, HILT aids in reducing pain and enhancing motor function in disk herniation with motor deficits, supporting neurological regeneration. It has shown efficacy in neurological recovery, particularly in lumbar disk herniation, by decreasing pain and improving neurological deficits. HILT has also been successfully applied in post-lung transplant recovery and pulmonary rehabilitation [58,59,60,61,62,63,64,65,66,67,68,69,70].

Kinesiotherapy aimed at increasing exercise capacity, such as resuming daily activities where appropriate, and enhancing overall muscle tone, with a focus on the diaphragmatic and accessory respiratory muscles was conducted. Techniques included respiratory re-education, functional neuromuscular proprioception (FNP) to increase joint range of motion, isotonic and isometric exercises (with or without weights) for global muscle training, and aerobic exercises to improve cardiovascular capacity and endurance. Kinesiotherapy sessions lasted between 30 and 60 min each day for 10 days. These sessions, conducted by a kinesiotherapist, included the following:

- Lung training: exercises to expand the chest and strengthen the diaphragm;
- Musculoskeletal system training: stretching exercises for the muscular and fascial chains;
- Strength training: exercises to tone various muscle groups;
- Balance training: static balance exercises on unstable surfaces and dynamic exercises such as walking over obstacles or resisting an elastic band;
- Aerobic training: therapeutic exercises using an ergometric bicycle, treadmill, or stepper.

The sessions were tailored to each patient’s physical condition.

### 2.3. Research Hypotheses

The study hypothesized that post-COVID rehabilitation significantly improves patients’ health and lifestyle by increasing effort tolerance and thereby facilitating their reintegration into society. This research aimed to highlight the impact of post-COVID rehabilitation on patient outcomes, objectively evaluated through functional testing that assesses both effort tolerance and walking capacity. The most commonly used test for this purpose is the 6MWT. To analyze the effectiveness of this test as a functional assessment tool in post-COVID rehabilitation, the study examined the impact of rehabilitation programs on the measured values of the test, correlating them with other clinical and biological assessments.

The study subjects were analyzed based on demographic data (age and sex) and biological and imaging data collected at admission. The evolution of functional parameters was monitored by comparing the values at admission with those at discharge.

Statistical analysis was performed using IBM SPSS Statistics 26 (IBM Inc., Chicago, IL, USA) and Excel 2021 Microsoft Office Professional Plus (Microsoft Corporation, Redmond, WA, USA). Descriptive analysis was conducted to summarize the data, and correlations between various parameters were tested to explore potential relationships and associations. Data normality was assessed, and a *p*-value threshold of *p* < 0.05 was set for statistical significance.

## 3. Results

The analyzed group consisted of 33 patients, 14 men and 19 women, aged between 30 and 80 years, 9% of them being smokers.

The study group consisted of four major classes of pathologies: pulmonary damage (post-COVID pneumonia with mild or moderate lung involvement, secondary pulmonary fibrosis), cardiac involvement (therapeutically controlled hypertension, NYHA class 2 heart failure with preserved ejection fraction, therapeutically controlled sinus tachycardia), neurological involvement (such as discogenic radiculopathies and polyneuropathies), and musculoskeletal involvement (discopathies, functionally decompensated knee osteoarthritis, and plantar fasciitis).

Regarding body mass index (BMI), 39.4% of patients were normal, 54.6% overweight and 6% obese, as shown in Table 1.

Regarding damages, 33.33% presented with pulmonary damage, 54.55% with musculoskeletal damage, 45.45% with neurological damage, and 21.21% with cardiac damage.

Fifty-five percent of patients underwent rehabilitation procedures that included HILT.

The participation rate for 6MWT was 97.06%. The functional assessment at admission and discharge is presented in Table 2.

Patients with a normal BMI performed better on the 6MWT than overweight and obese patients.

**Figure 1:**
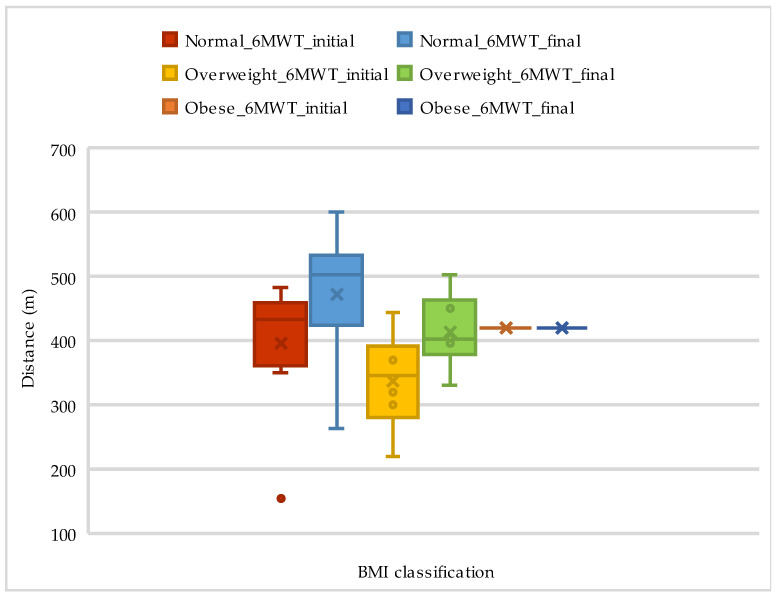
The evolution of walking distance on 6MWT according to BMI classification.

**Figure 2:**
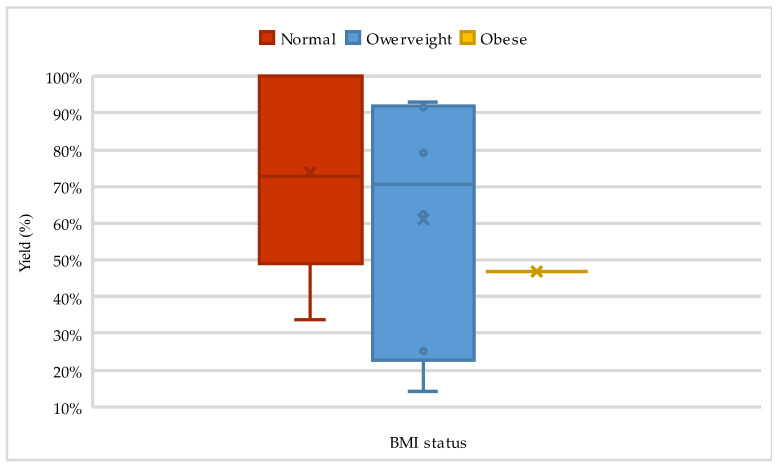
The evolution of 6MWT distance yield according to BMI classification.

Figure 1 and Figure 2 present data on walking distance from the 6MWT over time, comparing different BMI groups and showing the distance yield, based on BMI classification, as presented in Table 3.

**Figure 3:**
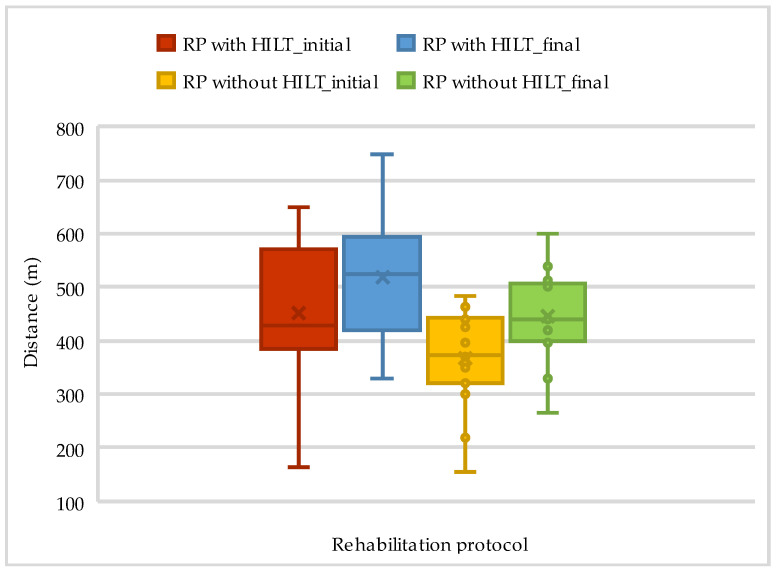
The evolution of walking distance on 6MWT according to rehabilitation protocol.

Out of the 33 patients, 18 underwent physiotherapy procedures that included HILT. The results of the 6MWT and Borg scale assessments at admission and discharge are presented in Table 4 and Figure 3.

**Figure 4:**
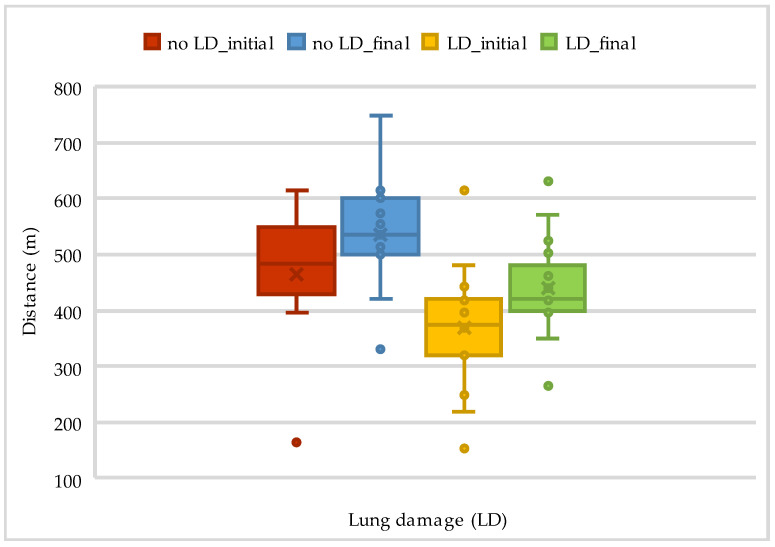
The evolution of walking distance on 6MWT according to lung damage.

The results of the 6MWT and Borg scale assessments at admission and discharge according to lung damage are presented in Table 5 and Figure 4.

**Figure 5:**
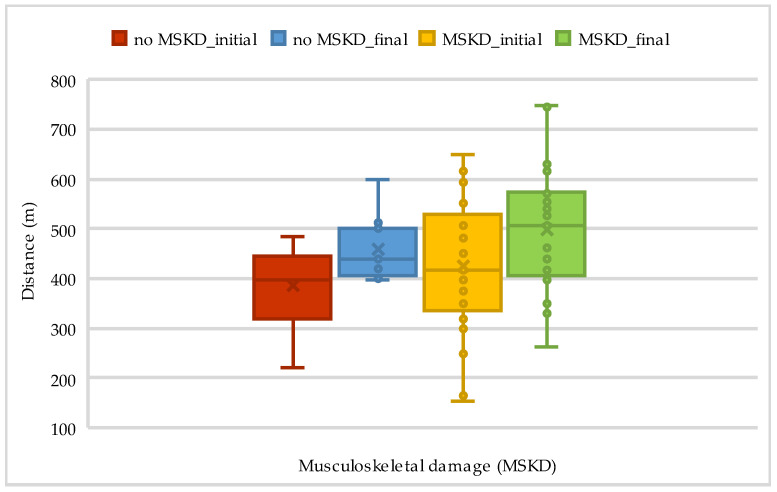
The evolution of walking distance on 6MWT according to musculoskeletal damage.

Out of 33 patients, 18 had musculoskeletal damage, which influenced the results of both the 6MWT and Borg exertion, as presented in Table 6 and Figure 5 below.

**Figure 6:**
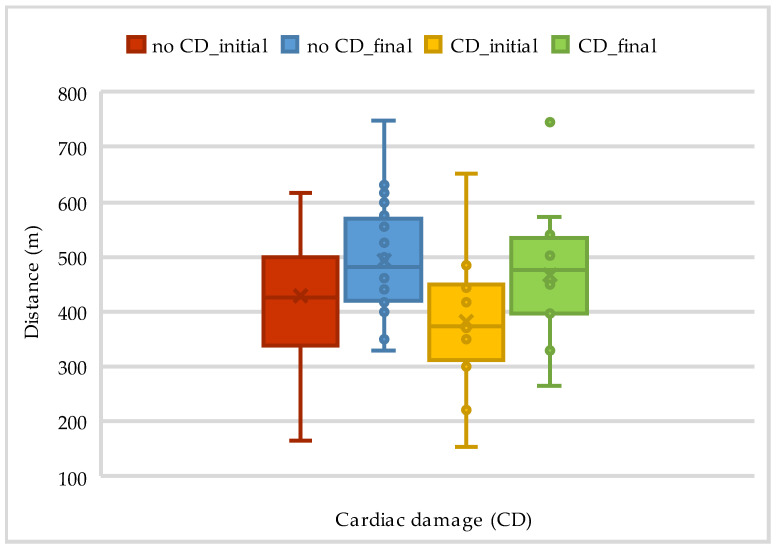
The evolution of walking distance on 6MWT according to cardiac damage.

Seven patients had cardiac damage, which affected the results of both the 6MWT and Borg exertion, as presented in Table 7 and Figure 6 below.

**Figure 7:**
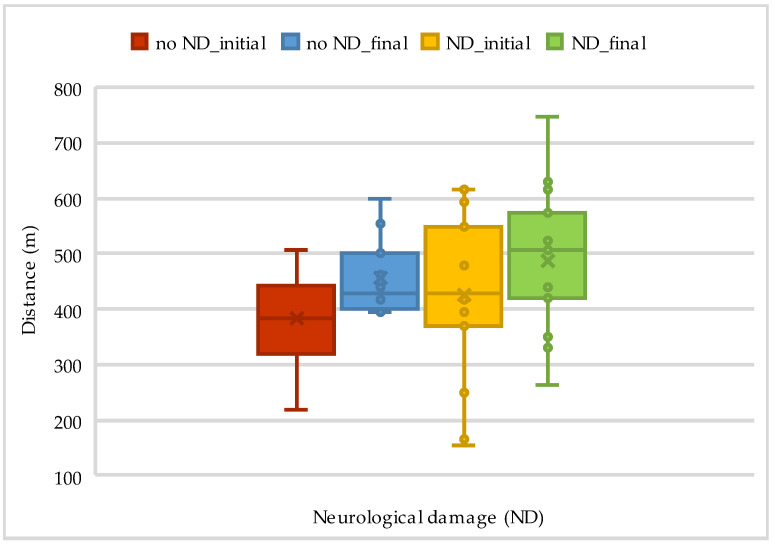
The evolution of walking distance on 6MWT according to neurological damage.

Out of 33 patients, 15 had neurological damage, which influenced the results of both the 6MWT and Borg exertion, as presented in Table 8 and Figure 7 below.

The statistical analysis indicated the presence of the following correlations:

- Age with walking distance at admission (*p* < 0.01, r = −0.636) and discharge (*p* < 0.01, r = −0.541);
- Age with oxygenation at admission (*p* < 0.01, r = −0.527) and discharge (*p* < 0.01, r = −0.552);
- Age with cardiac involvement (*p* < 0.01, r = 0.499);
- BMI with exertion at admission (*p* < 0.01, r = 0.506) and with lung damage (*p* < 0.01, r = 0.482);
- The 6MWT distance at admission with exertion at admission (*p* < 0.05, r = −0.403), lung damage (*p* < 0.05, r = −0.399), D-dimers (*p* < 0.01, r = −0.620), and fibrinogen (*p* < 0.01, r = −0.438);
- The 6MWT distance at discharge with 6MWT at admission (*p* < 0.01, r = 0.893), exertion at admission (*p* < 0.01, r = −0.449) and at discharge (*p* < 0.05, r = −0.362), with lung damage (*p* < 0.05, r = −0.459), and D-dimers (*p* < 0.01, r = −0.576);
- O2 saturation at discharge with O2 saturation at admission (*p* < 0.01, r = 0.613) and with cardiac damage (*p* < 0.01, r = −0.530);
- Exertion at admission with exertion at discharge (*p* < 0.01, r = 0.810), cardiac damage (*p* < 0.05, r = 0.389), and Fibrinogen (*p* < 0.05, r = 0.474);
- Exertion at discharge with cardiac damage (*p* < 0.05, r = 0.382);
- Lung damage with cardiac damage (*p* < 0.05, r = 0.430);
- Musculoskeletal damage with neurological damage (*p* < 0.05, r = 0.456);
- HILT with distance at discharge (*p* < 0.01, r = −0.692) and with yield distance (*p* < 0.01, r = −0.666);
- On patients with no HILT: 6MWT distance at admission with age (*p* < 0.01, r = −0.710), 6MWT distance at discharge with distance at admission (*p* < 0.01, r = 0.912), and age (*p* < 0.01, r = −0.585).

## 4. Discussion

This study investigated the correlations between various clinical parameters, rehabilitation interventions, and the 6-Minute Walk Test (6MWT) outcomes in post-COVID patients. The results provided important insights into the impact of rehabilitation, specifically the use of High-Intensity Laser Therapy (HILT), on functional recovery, the influence of age, BMI, and other factors, on walking capacity and exertion levels.

### 4.1. Impact of Age on Functional Outcomes

The analysis revealed significant negative correlations between age and walking distance at admission and discharge, with younger patients performing better on the 6MWT. These findings are consistent with the previous literature, which suggests that aging is associated with decreased physical performance and longer recovery times in post-illness rehabilitation [71,72]. Moreover, younger patients also showed better oxygenation at admission and discharge, which may be partially explained by the greater resilience of younger individuals in the face of pulmonary and systemic inflammation. The association between age and increased cardiac involvement aligns [73,74] with existing research indicating that elderly patients are more likely to experience severe cardiovascular complications during COVID-19 infection [75,76].

### 4.2. Influence of BMI on Exertion and Lung Damage

The correlation between BMI and exertion at admission as well as lung damage suggests that obese patients experienced greater difficulty during rehabilitation and were more affected by the lingering lung damage post-COVID. This is consistent with findings from the literature that indicate obesity as a risk factor for poorer outcomes in respiratory recovery [77,78,79]. The added strain of excess body weight may contribute to reduced physical endurance and a longer recovery time for both the acute and chronic phases of the illness.

### 4.3. Correlation Between 6MWT Performance and Inflammatory Markers

The results also demonstrated significant correlations between the 6MWT distance at admission and markers of inflammation, such as D-dimers and fibrinogen. Increased levels of D-dimers and fibrinogen have been identified as markers of ongoing systemic inflammation, which is known to persist even after acute COVID-19 symptoms resolve [80,81,82,83,84]. These findings underscore the importance of considering inflammation, even when asymptomatic, as a contributing factor to impaired functional capacity. The persistence of inflammation was also reflected in the 6MWT distance at discharge, which correlated with D-dimer levels. This suggests that ongoing inflammation, particularly in the lungs, can significantly influence walking capacity, which is crucial for assessing long-term recovery and the effectiveness of rehabilitation programs.

### 4.4. Role of HILT in Rehabilitation

HILT was found to have a significant positive impact on the 6MWT distance at discharge and on the yield of the distance. These results suggest that HILT played an important role in improving walking capacity and functional recovery in post-COVID patients. Previous studies have demonstrated that laser therapy can aid in tissue repair and reduce inflammation, which could explain its beneficial effects on recovery from post-viral sequelae [85]. This is particularly relevant in the context of post-COVID rehabilitation, where managing persistent inflammation and promoting tissue healing are critical to improving overall functional capacity.

### 4.5. Musculoskeletal and Neurological Impacts

The correlation between musculoskeletal and neurological damage further highlights the widespread effects of COVID-19 on the peripheral nervous and musculoskeletal systems. This finding supports the notion that COVID-19 can have a multifaceted impact on the neuromuscular and musculoskeletal systems, leading to long-lasting impairments in mobility and functional independence [86,87,88,89,90]. The evidence from this study emphasizes the importance of comprehensive rehabilitation programs that address physical and neurological rehabilitation to optimize recovery.

### 4.6. The 6MWT as a Sensitive Indicator of Recovery

The 6MWT proved to be a sensitive and comprehensive indicator of patient recovery across a wide range of clinical variables. It correlated with nearly all parameters studied, including demographic factors (age and BMI), comorbidities, D-dimers, fibrinogen, HILT interventions, and other clinical markers. The 6MWT is an effective tool for assessing current functional status and comparing results with predicted values before and after a rehabilitation program, offering valuable insights for clinicians managing post-COVID rehabilitation.

Furthermore, the high participation rate (97.06%) in the 6MWT demonstrates its acceptability and ease of use among patients. This is crucial, as patient compliance is often a limiting factor in implementing rehabilitation protocols. Given its wide applicability and ability to reflect the impact of the disease and rehabilitation interventions, the 6MWT can serve as a key functional measure in post-COVID care.

### 4.7. Variation in Perceived Exertion During Physical Activity

At admission, perceived exertion varied widely, ranging from 0 to 8, indicating that some patients experienced no exertion while others felt a moderate to high level of effort during physical activities. This variability suggests differing initial physical conditions and endurance levels among the patients, which could be attributed to individual factors such as baseline fitness, severity of post-COVID symptoms, or the presence of musculoskeletal comorbidities. Upon discharge, scores improved across the group, with perceived exertion ranging from 0 to 5. This reduction in Borg scale scores at discharge indicates that patients, on average, experienced less strain during physical activities after completing the rehabilitation program. This outcome suggests an improvement in physical tolerance and a decrease in the effort required for daily activities, potentially due to the combined effects of High-Intensity Laser Therapy (HILT) and kinesiotherapy in promoting recovery. These findings highlight the effectiveness of rehabilitation in reducing physical effort and increasing functional capacity among post-COVID patients. Future studies could explore whether incorporating more individualized physical and psychological support strategies might further optimize these outcomes, particularly for patients starting with higher Borg scores.

When analyzing perceived exertion (measured via the Borg scale) across different BMI categories, distinct patterns emerge, shedding light on the impact of body mass on physical endurance and recovery progression during rehabilitation. These findings underscore the variability of perceived exertion among BMI categories and emphasize the need for tailored rehabilitation protocols. Patients with higher BMI values may initially experience more difficulty with physical tasks, but the observed improvements by discharge suggest that a structured rehabilitation program can significantly alleviate this effort.

In patients with musculoskeletal disorders treated with HILT at admission, the Borg scale scores ranged from 0.5 to 5, indicating a moderate level of perceived exertion. By discharge, scores had decreased to a range between 0 and 3, reflecting a significant reduction in perceived effort. This improvement suggests that HILT, combined with other rehabilitation therapies, may have effectively alleviated pain or discomfort associated with musculoskeletal conditions, allowing these patients to engage in physical activity with less strain over time. Patients without HILT treatment showed a broader range of perceived exertion, with Borg scale scores spanning from 0 to 8 at admission. By discharge, scores decreased to a range between 0 and 5, indicating some improvement but still showing a higher perceived effort level on average compared to those treated with HILT. The larger initial and discharge ranges suggest greater variability in physical endurance and exertion levels among this group, possibly due to differences in baseline health conditions or the absence of targeted interventions like HILT.

These findings suggest that comorbidities such as lung, cardiac, and neurological damage play a role in patients’ perceived exertion and response to rehabilitation. Cardiac damage, in particular, appears to correlate with higher exertion at discharge, possibly due to the additional cardiovascular strain. Meanwhile, patients with lung and neurological issues showed substantial improvements, though with slightly higher discharge scores than those without these conditions. Tailoring rehabilitation interventions to address specific comorbidities may further optimize recovery outcomes, potentially focusing on cardiovascular support for patients with cardiac damage and endurance training for those with lung and neurological impairments.

### 4.8. Limitations and Further Research

While the data from this study provide valuable insights into the rehabilitation of post-COVID patients, several limitations must be acknowledged. The small sample size, particularly among smokers, limited the ability to detect significant correlations in this subgroup. Additionally, this study did not account for potential psychosocial factors that could influence functional outcomes. Future studies with larger, more diverse populations and a broader range of variables, including mental health and quality of life measures, will provide a more comprehensive understanding of post-COVID recovery and the role of rehabilitation interventions like HILT. We also aim to conduct a prospective study on the use of HILT in musculoskeletal conditions with a larger group of patients.

## 5. Conclusions

This study demonstrates that the 6-Minute Walk Test (6MWT) is a sensitive and reliable tool for assessing functional capacity and monitoring the effects of inflammation in post-COVID patients. Post-COVID rehabilitation, including interventions such as High-Intensity Laser Therapy (HILT), plays a critical role in improving functional outcomes, like walking distance and exertion tolerance. HILT, when combined with a kinetic rehabilitation program, can effectively address post-COVID sequelae. These findings underscore the importance of personalized rehabilitation programs that consider patient-specific factors, such as age, BMI, and comorbidities, to optimize recovery and enhance quality of life in this population. 

Further research is needed to explore the long-term impacts of rehabilitation interventions and deepen our understanding of the complex recovery process following COVID-19.

## Figures and Tables

**Table 1:** Characteristics of the study group participants.

	HILT		No HILT	
No	18		15	
Sex—Male	7		7	
Age (yrs.) Min ÷ Max	30 ÷ 72		41 ÷ 79	
BMI classification				
Normal	5		8	
Overweight	12		6	
Obese	1		1	
Comorbidities				
	yes	no	yes	no
Musculoskeletal damage	18	0	0	15
Lung damage	10	8	8	7
Neurological damage	2	16	5	10
Cardiac damage	11	7	4	11

**Table 2:** Functional assessment at admission and discharge.

Parameter	Admission	Discharge
6MWT Min ÷ Max % from ideal walking distance	154 ÷ 650 36 ÷ 98%	264 ÷ 748 56 ÷ 100%
O2 saturation Min ÷ Max	91 ÷ 99	93 ÷ 99
Borg exertion Min ÷ Max	0 ÷ 8	0 ÷ 5

**Table 3:** The 6MWT and Borg exertion scale based on BMI classification.

BMI Classification	Values	6 MWT		Exertion (Borg)	
		Admission	Discharge	Admission	Discharge
Normal	Min ÷ Max Mean	154 ÷ 650 437.62	264 ÷ 745 505.77	0 ÷ 3 1.42	0 ÷ 1 0.38
Overweight	Min ÷ Max Mean	165 ÷ 616 398	330 ÷ 748 476.12	0.5 ÷ 8 2.88	0 ÷ 5 1.24
Obese	Min ÷ Max Mean	320 ÷ 396 358	420	3 ÷ 4 3.5	0

**Table 4:** The 6MWT and Borg exertion according to rehabilitation procedures.

Rehabilitation Procedures	Values	6 MWT		Exertion (Borg)	
		Admission	Discharge	Admission	Discharge
With HILT	Min ÷ Max Mean	165 ÷ 650 450.71	330 ÷ 748 519	0.5 ÷ 5 2.03	0 ÷ 3 0.71
Without HILT	Min ÷ Max Mean	154 ÷ 484 367.27	264 ÷ 600 445.73	0 ÷ 8 2.67	0 ÷ 5 0.93

**Table 5:** The 6MWT and Borg exertion according to lung damage.

Lung Damage (LD)	Values	6 MWT		Exertion (Borg)	
		Admission	Discharge	Admission	Discharge
No LD	Min ÷ Max Mean	165 ÷ 616 466	330 ÷ 748 536.18	0.5 ÷ 5 1.86	0 ÷ 2 0.55
LD	Min ÷ Max Mean	154 ÷ 396 370.29	264 ÷ 462 440.58	0.5 ÷ 5 2.47	0 ÷ 3 0.82

**Table 6:** The 6MWT and Borg exertion according to musculoskeletal damage.

Musculoskeletal Damage (MSKD)	Values	6 MWT		Exertion (Borg)	
		Admission	Discharge	Admission	Discharge
No MSKD	Min ÷ Max Mean	220 ÷ 484 386.82	396 ÷ 600 458.73	1 ÷ 2 2.36	0 ÷ 1 0.64
MSKD	Min ÷ Max Mean	154 ÷ 418 424.57	264 ÷ 572 498.24	0.5 ÷ 5 2.3	0 ÷ 3 0.71

**Table 7:** The 6MWT and Borg exertion according to cardiac damage.

Cardiac Damage (CD)	Values	6 MWT		Exertion (Borg)	
		Admission	Discharge	Admission	Discharge
No CD	Min ÷ Max Mean	165 ÷ 616 429.15	330 ÷ 748 494.50	0.5 ÷ 5 1.98	0 ÷ 3 0.55
CD	Min ÷ Max Mean	154 ÷ 444 336.29	264 ÷ 572 426.43	2 ÷ 5 3.14	1 ÷ 3 1.29

**Table 8:** The 6MWT and Borg exertion according to neurological damage.

Neurological Damage (ND)	Values	6 MWT		Exertion (Borg)	
		Admission	Discharge	Admission	Discharge
No ND	Min ÷ Max Mean	220 ÷ 506 383.42	396 ÷ 600 457.83	1 ÷ 5 3	0 ÷ 3 1
ND	Min ÷ Max Mean	154 ÷ 616 426.80	264 ÷ 748 488.13	0.5 ÷ 4 1.63	0 ÷ 2 0.47

## Data Availability

The corresponding authors can provide access to the data contained in this study upon request.
